# Shifts in Mycobacterial Populations and Emerging Drug-Resistance in West and Central Africa

**DOI:** 10.1371/journal.pone.0110393

**Published:** 2014-12-10

**Authors:** Florian Gehre, Mebrat Ejo, Kristina Fissette, Pim de Rijk, Cécile Uwizeye, Elie Nduwamahoro, Odin Goovaerts, Dissou Affolabi, Martin Gninafon, Fanny M. Lingoupou, Mamadou Dian Barry, Oumou Sow, Corinne Merle, Piero Olliaro, Fatoumata Ba, Marie Sarr, Alberto Piubello, Juergen Noeske, Martin Antonio, Leen Rigouts, Bouke C de Jong

**Affiliations:** 1 Institute of Tropical Medicine (ITM), Antwerp, Belgium; 2 Medical Research Council (MRC), The Gambia Unit, Fajara, The Gambia; 3 Laboratoire de Reference des Mycobactéries, Cotonou, Benin; 4 Laboratoire des Mycobactéries, Institut Pasteur de Bangui, Bangui, Central African Republic; 5 Laboratoire de Reference des Mycobactéries, Conakry, Guinea-Conakry; 6 London School of Hygiene and Tropical Medicine, London, United Kingdom; 7 UNICEF/UNDP/World Bank/WHO Special Programme for Research and Training in Tropical Diseases (TDR), Geneva, Switzerland; 8 Laboratoire de Reference des Mycobactéries, Dakar, Senegal; 9 National TB Programme, Dakar, Senegal; 10 Damien Foundation, Niamey, Niger; 11 Independent Consultant, Yaoundé, Cameroon; 12 New York University, New York, United States of America; University of Florence, Italy

## Abstract

In this study, we retrospectively analysed a total of 605 clinical isolates from six West or Central African countries (Benin, Cameroon, Central African Republic, Guinea-Conakry, Niger and Senegal). Besides spoligotyping to assign isolates to ancient and modern mycobacterial lineages, we conducted phenotypic drug-susceptibility-testing for each isolate for the four first-line drugs. We showed that phylogenetically modern *Mycobacterium tuberculosis* strains are more likely associated with drug resistance than ancient strains and predict that the currently ongoing replacement of the endemic ancient by a modern mycobacterial population in West/Central Africa might result in increased drug resistance in the sub-region.

## Introduction

Tuberculosis is one of the most important infectious diseases worldwide. In 2012, the World Health Organization (WHO) estimated 8.6 million newly infected cases and 1.3 million deaths globally, with Asia and Africa carrying the major burden of disease. Moreover, as of 2012, Africa did not reach WHO mortality and prevalence targets, and the emergence of drug resistance is a pressing public health concern [1].

The advent of modern single nucleotide polymorphism (SNP)-based phylogeny not only lead to a conclusive classification of the *Mycobacterium tuberculosis* complex (MTBC) into seven major human lineages, but also demonstrated that Africa was the geographic origin of all known lineages and the global TB pandemic [2]. Several authors have shown that phylogenetically distinct “modern” strains (lineages 2, 3 and 4 – also known as East Asian, East-African Indian and Euro-American lineages) branched off from the existing “ancient” African strains (lineages 1, 5, and 6 – also known as Indo-Oceanic lineage, *M. africanum* West Africa 1 and 2) and were globally dispersed paralleling the human out-of-Africa migration [2], [3]. While active transmission of the two ancient *M. africanum* lineages has only been detected in West Africa, modern lineages spread around the world and were later re-introduced into Africa with arrival of the colonial powers [2].

West-, and parts of Central Africa, indeed have an intriguing mycobacterial population structure, as they are the only regions worldwide where all three ancient lineages 1, 5 and 6 are endemic. However, recent publications from various countries observed an interesting trend: the replacement of ancient lineages with modern strains. This phenomenon was first described in Guinea-Bissau, where *M. africanum* West African 2 decreased from 51% to 39% between 1989–2008 (19 years), supported by declines in prevalence of *M. africanum* lineages observed in Côte d’Ivoire, Ghana and Cameroon, amongst others [4], [5], [6], [7]. The speed of this replacement is remarkable in evolutionary terms, as mere decades ago ancient lineages contributed up to half of all TB cases in some areas [4], [5], where prevalence has now declined to a small proportion of all cases [5].

We hypothesized that this major bacterial population shift may impact on TB control in the sub-region. Such an assumption is reasonable as lineages/families tend to vary in phenotypes, potential to transmit, or ability to cause disease [8], [9], [10]. As drug resistance is of major public health relevance, we focused on the question whether ancient strains are less likely associated with drug resistance, as has been suggested for lineage 1 in India [11]. If so, then the presently ongoing transition towards modern strains might result in increased drug-resistance in West and Central Africa.

## Materials and Methods

### Sample collection

The samples analysed within this retrospective study had drug-susceptibility testing (DST) done on arrival, prior to storage at the Institute of Tropical Medicine (ITM) in Antwerp and originated from various study sites and time periods (see Table 1 and S1 Table for detailed information). While samples from Benin, Senegal and Guinea-Conakry were collected as part of the Oflotub study, samples from Cameroon, Niger and the Central African Republic were collected for quality assurance by the ITM supra-national Reference Laboratory. Ethics permissions were obtained from the ITM Institutional Review Board.

**Table 1:** Geographical origin and treatment history of the included patient samples (n = 605).

Country	Treatment history		
	New cases	Retreatment cases	Total
	no. (%)	no. (%)	no. (%)
Benin	43 (9.7%)	43 (26.5%)	86 (14.2%)
Cameroon	145 (32.7%)	20 (12.3%)	165 (27.3%)
Central African Republic	102 (23%)	-	102 (16.9%)
Guinea Conakry	120 (27.1%)	-	120 (19.8%)
Niger	-	53 (32.7%)	53 (8.8%)
Senegal	33 (7.4%)	46 (28.4%)	79 (13.1%)
Total	443 (100%)	162 (100%)	605 (100%)

### Drug-susceptibility testing (DST)

To determine the drug resistance profile of each isolate we applied phenotypic drug-susceptibility testing (DST) using the proportion method. Isolates were grown at 37°C on Löwenstein-Jenssen slopes containing either rifampicin (RMP) 1 µg/ml, isoniazid (INH) 0.2 µg/ml, ethambutol (EMB) 2 µg/ml or streptomycin (SM) 4 µg/ml [12],[13].

### Spoligotyping and lineage assignment of isolates

To classify isolates into lineages, boiled culture lysates were prepared and spoligotyped as described elsewhere [14]. Based on the binary spoligotype codes, lineage assignment to distinguish modern from ancient strains was performed using the publicly available online package “TBLineage” [15]. The spoligotype international identifier (SIT) number was assigned using SITVITweb (http://www.pasteur-guadeloupe.fr:8081/SITVIT_ONLINE/) [16].

### Statistical analysis

As differing sampling protocols resulted in the inclusion of isolates from patients with differing treatment history (new/retreatment cases) (see Table 1), we adjusted for that variable in any analysis conducted. To determine the association between mycobacterial lineage and drug-susceptibility we conducted a logistic regression and calculated the Mantel-Haenszel Odds Ratio (OR) and 95% Confidence Intervals, adjusted for treatment history of patients.

## Results and Discussion

Six hundred and five MTBC isolates of new and retreatment cases, from two Central and four West African countries were included into this study (Table 1). A detailed description of each isolate, its origin, time of sampling, DST profile and spoligotype international type (SIT) can be found in S1 Table. Although treatment history of patients was not a significant confounder in our dataset (data not shown), we still considered it in our final analysis. Combining obtained spoligotyping and DST data, and conducting a multivariate analysis adjusted for treatment history (new/retreatment cases), we found a strong association between lineage and drug resistance (Table 2). Specifically, strains belonging to modern lineages were more likely resistant to any of the tested drugs compared to ancient strains (adjusted Odds Ratio (OR), 95%CI = 3.20 (1.56–6.54), p = 0.0008). When analysing results by individual drugs, we found that this result was mainly driven by the higher risk of resistance towards INH and SM (adjusted OR 95%CI = 2.71 (1.27–5.77), p = 0.0073 for INH and adjusted OR 95%CI = 2.69 (1.23–5.85), p = 0.0095 for SM) (Table 2). The association was not demonstrated for RMP and EMB, nor for multi-drug-resistant TB (MDR-TB) isolates, which constituted 17.7% of the total sample.

**Table 2:** Association between modern (lineages 2, 3, 4) and ancient (lineage 1, 5, 6) mycobacterial lineages and drug-susceptibility testing (DST), overall resistance and stratified by individual drugs.

	Total no. (%)	Modern no. (%)	Ancient no. (%)	Adjusted Odds RatioOR (95% CI)*	p-value
***DST profile***					
Any resistance	226 (62.6%)	214 (39.5%)	12 (19%)	**3.20 (1.56–6.54)**	**0.0008**
***Resistance to individual drugs***					
Isoniazid	178 (29.4%)	168 (31.0%)	10 (15.9%)	**2.71 (1.27–5.77)**	**0.0073**
Ethambutol	85 (14.0%)	79 (14.6%)	6 (9.5%)	1.80 (0.68–4.74)	0.2286
Rifampicin	112 (18.5%)	104 (19.2%)	8 (12.7%)	1.78 (0.79–4.01)	0.1574
Streptomycin	177 (29.3%)	167 (30.1%)	10 (15.9%)	**2.69 (1.23–5.85)**	**0.0095**
MDR	107 (17.7%)	99 (18.3%)	8 (12.7%)	1.66 (0.74–3.74)	0.2130
Total	605 (100%)	542 (100%)	63 (100%)	-	-

*Mantel-Haenszel OR adjusted for treatment history of patients (new/retreatment case).

Although previous publications demonstrated an association between lineage and drug-susceptibility in other parts of the world before [11], it was not known whether these results can be generalized and extrapolated to West and Central Africa and its geographically highly restricted ancient mycobacterial population that mainly consists of *M. africanum* isolates. Interestingly, our results confirmed and reproduced these recent findings and demonstrated that the introduced modern strains have increased potential to develop INH- and SM-resistance, when compared to the endemic ancient African lineages. Replacement of “susceptible” ancient strains by resistant modern strains will thus likely lead to higher prevalence of resistant mycobacteria. This is an important finding especially for public health practitioners in West and Central Africa. Although ∼10% of the analyzed isolates in the current study were ancient strains, suggesting that the lineage replacement has already taken place, there are several West African countries, such as Gambia, Guinea-Bissau, Ghana, Sierra Leone, Mali and Nigeria, in which ancient strains remain an important cause of tuberculosis [4], [7], [9], [10], [17], [18], [19], [20]. In these very settings *M. africanum* can still be responsible for up to one third of pulmonary TB, and further replacement by modern *M. tuberculosis*, with an increased risk of resistance to at least one drug (adjusted OR 3.2, Table 2), may seriously complicate TB case management and control strategies. For instance, the predicted elevated INH resistance prevalence and the use of INH and RMP in the continuation phase of current shortcourse treatment for 4 months might ultimately result in increased RMP resistance as well. Although we have not found any association with MDR-TB yet, probably due to the limited number of ancient strains and lack of statistical power, INH resistance was already shown to increase the likelihood of acquiring additional RMP resistance before [21]. In addition, systematically treating patients with treatment failure with SM in absence of DST might not be appropriate if prevalence of SM resistance is increasing.

Although we successfully confirmed the described association between ancient strains and drug-susceptibility [11] in our research area in West and Central Africa, possible limitations of our study include the retrospective approach and the low proportion of identified ancient lineages. To conclusively demonstrate causation between mycobacterial lineage and drug-resistance a prospective longitudinal large-scale multi-centered study needs to be conducted.

The reason for the observed emergence of modern strains is elusive, however it was found that these lineages progress to disease faster [8]. This was hypothesized to provide an adaptation to the overcrowding of humans in cities, in which new susceptible hosts are in abundance and there is no selective advantage to delay progression until a new generation of hosts becomes available. Due to the increased urbanization in Africa, modern strains may have a selective advantage over ancient, “rural” strains. Another explanation could be that modern strains are more likely to develop drug resistance and the increased roll-out of drug-based TB control programs in West and Central Africa, with higher levels of drug pressure, could potentially select for resistant lineages. Similarly, it could be conceivable that BCG vaccination is more efficiently protecting against ancient than modern mycobacterial lineages, due to the closer phylogenetic relatedness of these ancient lineages to the *M. bovis* BCG vaccine strains [22]. However, no such correlation was found in previous studies from The Gambia [23] and since the present dataset was limited to bacterial data and did not include the BCG status of the patients, we were not able to test this hypothesis in the present study.

Independent of exact mechanisms triggering the replacement of ancient by modern strains, our findings predict that ongoing mycobacterial population dynamics might accelerate the emergence of antibiotic-resistance and should be considered by public health professionals when planning and evaluating drug resistance control programs in West and Central Africa.

## Supporting Information

Table S1 **Genotypic and DST information on all isolates included in the study.**
(XLSX)
